# Supplementary material for: Effect of Inappropriate Treatment in Hospitalized Patients with Pyelonephritis Treated with Cefuroxime: A Cohort Study
Source: Antibiotics (Basel). 2024 Mar 19;13(3):274. doi: 10.3390/antibiotics13030274 (PMC10967530; doi:10.3390/antibiotics13030274)
Supplement: Supplementary file 1 [file antibiotics-13-00274-s001.zip › antibiotics-2862832-supplementary.pdf]

Supplementary material to **Jorge Alberto Cortés [1,*], Ricardo Sánchez [2] and Claudia Rocío Sierra [3], Effect of Inappropriate Treatment in Hospitalized Patients with Pyelonephritis treated with Cefuroxime in a Cohort Study**

Table S1. Susceptibility results from *E. coli* isolates from community acquired pyelonephritis.

Antibiotic	Number tested	Susceptibility (%)
Ampicillin	727	41.1
Ampicillin-sulbactam	747	49.1
Cefazolin	726	55.8
Cefuroxime	747	86.3
Ceftriaxone	746	89.8
Cefotaxime	726	90.1
Ceftazidime	747	94.8
Cefepime	746	97.0
Ertapenem	745	100.0
Meropenem	747	100.0
Ciprofloxacin	746	70.2
Amikacin	747	98.8
Gentamicin	744	84.7

Table S2. Parametric model (Weibull) for the association between inappropriate therapy and length of stay using a matched propensity score.

Variable	HR	IC95%
Inappropriate therapy	0.26	0.12 – 0.54
Oral treatment at discharge	0.14	0.08 – 0.24
One or more comorbidities	2.22	1.58 – 3.13
More than 60 years-old	1.36	0.95 – 1.97
Antibiotic change during stay	3.28	1.48 – 7.29
ICU admission	6.59	3.2 – 13.6

Data from 320 patients, matched 3:1 (105 patients with exposure).

Table S3. Association between inappropriate therapy and length of stay using a Poisson model.

Variable	aIRR*	IC95%
Inappropriate therapy	0.56	0.46 – 0.70
Oral treatment at discharge	0.61	0.54 – 0.69
One or more comorbidities	1.13	1.04 – 1.21
More than 60 years-old	1.15	1.07 – 1.23
Antibiotic change during stay	1.54	1.28 – 1.86
ICU admission	1.40	1.21 – 1.62

*aIRR: adjusted incidence rate ratio
